# Effect of Regular Taekwondo Self-Defense Training on Oxidative Stress and Inflammation Markers in Postmenopausal Women

**DOI:** 10.3390/healthcare9080985

**Published:** 2021-08-03

**Authors:** Beom-Jun Ku, Kangeun Ko, Ki-Ok Shin, Ju-Yong Bae

**Affiliations:** 1 Department of Taekwondo, College of Arts and Physical Education, Dong-A University, Busan 49315, Korea; kbj72621@naver.com; 2 Laboratory of Exercise Biochemistry, Department of Physical Education, College of Arts and Physical Education, Dong-A University, Busan 49315, Korea; kku0803@naver.com

**Keywords:** antioxidant enzymes, exercise, lipid profile, martial arts, physical fitness, reactive oxygen species

## Abstract

We aimed to investigate the effect of a 12-week Taekwondo self-defense training course on oxidative stress and inflammation in postmenopausal women. Sixteen middle-aged women participated and were randomized into two groups: a control group (CG, *n* = 8) and a Taekwondo self-defense training group (TSDG, *n* = 8). The TSDG was trained for 60 min, four times per week, for 12 weeks. Following the Taekwondo training intervention, side-step was significantly higher in the TSDG than in the CG (*p* < 0.001). Malondialdehyde levels were significantly lower after the intervention than before in the TSDG (*p* < 0.01). Superoxide dismutase (SOD) levels were also significantly higher after the intervention than before in the TSDG (*p* < 0.001). After the Taekwondo training intervention, SOD levels were significantly higher in the TSDG than in the CG (*p* < 0.01). Tumor necrosis factor α (TNF-α) levels were significantly lower after the intervention than before in the TSDG (*p* < 0.05). After the Taekwondo training intervention, TNF-α levels were significantly lower in the TSDG than in the CG (*p* < 0.05). The results of this study suggest that Taekwondo self-defense training is an effective exercise that improves agility, oxidative stress, and inflammatory responses in postmenopausal women.

## 1. Introduction

Middle-aged women experience menopausal symptoms, such as anxiety, depression, and insomnia, due to changes in hormone secretion. The risks of cardiovascular disease and lifestyle-related diseases, such as obesity, high blood pressure, and diabetes, are also increased [1,2]. Overproduction of reactive oxygen species (ROS) promotes age-related diseases, increasing the burden of psychological and physiological changes in postmenopausal women [3]. Malondialdehyde (MDA), a representative indicator of ROS, is a lipid peroxide that can damage cell membranes and macromolecules [4]. In contrast, to prevent excessive damage to cells due to ROS, antioxidant enzymes act as a defense system by reducing ROS to hydrogen peroxide and water through the generation of superoxide dismutase (SOD) and glutathione peroxidase (GPx) within cells [5]. Although the production of ROS and activities of the antioxidant enzymes are balanced in a healthy state, various stimuli can induce overproduction of ROS, leading to a reduction in antioxidant production. This in turn results in an imbalance in the oxidation–antioxidant system, otherwise referred to as oxidative stress [6]. This oxidative stress may induce an increased level of inflammatory markers including interleukin-6 (IL-6) and tumor necrosis factor α (TNF-α). As oxidative stress is closely related to the onset of disease, reduction in oxidative stress is important [7].

Regular physical exercise of moderate intensity is known to reduce oxidative stress-induced diseases by stimulating antioxidant defense systems, such as SOD and GPx [8]. Moreover, light to moderate intensity physical activity significantly increases GPx activity and decreases plasma oxidative stress in active compared to sedentary postmenopausal women [9]. In a study that analyzed the relationship between oxidative stress biomarkers and physical exercise in middle-aged men and women without cardiovascular disease, walking, dancing, and yoga showed decreased MDA levels, while walking and dancing were seen to be associated with increased SOD activity [10]. In addition, the level of oxidative stress markers improves as the frequency of exercise increases [10]. Thus, regular exercise helps improve the health of postmenopausal women by positively regulating the levels of their oxidative stress.

Taekwondo is a traditional Korean martial art which has been adopted as an official Olympic sport. To date, 207 countries have joined the World Taekwondo Federation [11]. Traditional forms of Taekwondo training are typically divided into poomsae and gyeorugi styles. While poomsae is a style that can be performed on the basis of pre-determined patterns and movements, gyeorugi, the official style performed in the Olympics, is the sparring form [12]. Various studies have shown that traditional Taekwondo training improves the health index of middle-aged adults. Chronic Taekwondo training has been shown to improve balance control in middle-aged adults aged >40 years [13]. In another study, a 16-week Taekwondo training program improved cognitive function in women aged >65 years [14]. In addition, Taekwondo training positively improved blood pressure levels and arterial stiffness in post-menopausal women with hypertension [15]. Thus, Taekwondo training has been proven to have a positive physiological effect on postmenopausal women.

In recent years, Kukkiwon has been striving to popularize new types of training methods such as Taekwondo gymnastics and Taekwondo self-defense, in addition to Taekwondo poomsae and gyeorugi. Moreover, as violent crimes increase in modern society, protecting oneself is becoming increasingly important. Despite the need for self-defense training, such training studies are scarce, and most have focused on the physical and psychological effects [16]. Our hypothesis was that Taekwondo self-defense training has additional effects on physiological changes in postmenopausal women, such as those related to oxidative stress and inflammation. Therefore, this study aimed to investigate the effect of a 12-week of regular Taekwondo self-defense training on oxidative stress and inflammation in postmenopausal women.

## 2. Materials and Methods

### 2.1. Subjects

Twenty healthy menopausal women aged >45 years with no medical conditions participated in this study. Using the G*Power test (G* Power 3.1.9.7, Heinrich-Heine-University, Düsseldorf, Germany), the number of subjects required was calculated to be 14 (effect size (ES) = 0.40, α value = 0.05, and desired statistical power (1-β) = 0.80). The subjects were randomly classified into the control group (CG, *n* = 10) or the Taekwondo self-defense training group (TSDG, *n* = 10).

The eligibility criteria included (1) age > 45 years, (2) diagnosis of menopause through blood test, (3) no ongoing hormone replacement therapy, (4) no current participation in any regular exercise program, and (5) no history of musculoskeletal or cardiovascular disorders. The exclusion criteria were (1) failure to meet the criteria for participation in exercise intervention (less than 90% attendance) and (2) inability to continue exercise due to musculoskeletal disorders.

**Figure 1:**
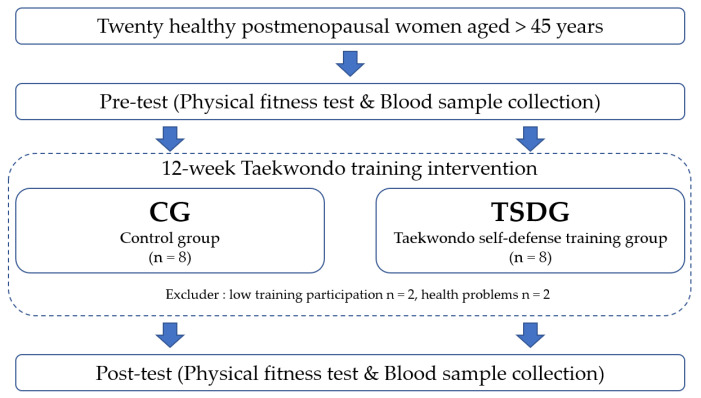
The experimental design.

Eight individuals in each group were included in the final analyses, following exclusion of four individuals who had low training participation (*n* = 2) or who developed problems with their health (*n* = 2). Prior to the start of the study, participants were informed about the purpose, method, and procedure of this study, and all provided written informed consent to participate. This study was approved by the Institutional Review Board of Dong-A University (IRB No. 2-1040709-AB-N-01-201911-BR-017-06) All procedures were conducted in accordance with the 1975 Declaration of Helsinki. The specific experimental design is presented in Figure 1, and the characteristics of the study subjects are listed in Table 1.

### 2.2. Physical Fitness Test

The physical fitness test was conducted before and after the 12 weeks of Taekwondo training intervention. The specific measurements of physical fitness that were measured using a body composition analyzer before and after the 12 week training intervention were as follows: Body composition parameters such as height (cm), weight (kg), fat mass (kg), percent fat (%), and lean body mass (kg). Body mass index (BMI) was calculated using the formula weight/height (kg/m^2^).

Lower back strength, side-step, trunk flexion, and Sargent jump were performed twice in correct postures after full explanation of the accurate measurement methods. Exercises conducted in incorrect postures were not included in the results. Measurements were performed in reference to a previous study [17], and an improved record was used as a result. Briefly, lower back strength was measured to evaluate muscular strength with 0.1 kg accuracy. A side-step test was performed to evaluate agility, and the test was carried out for 20 s on a mat measuring 120 cm from the left and right around the centerline. Trunk flexion was conducted to evaluate flexibility with a 0.1 accuracy. The Sargent jump was conducted to evaluate power with an accuracy of 0.1. The measuring equipment for each physical fitness component is presented in Table 2.

### 2.3. Taekwondo Training

The Taekwondo self-defense training program included warm-up exercises (10 min), main exercises (40 min), and cool-down exercises (10 min), totaling 60 min. The training was conducted four times a week for 12 weeks (Table 3). Taekwondo training was conducted in a group following the leader’s demonstrations and commands. The intensity of Taekwondo training was maintained by having participants wear a M430 device (Polar Electro Oy, Kempele, Finland), and the training intensity was set at 50–70% of the heart rate reserve with the subjects maintaining this range until the end of the intervention.

The Taekwondo self-defense training comprised self-defense exercises adopted from the International Taekwondo Master Course (3rd class) textbook published by Kukkiwon [18]. Movement consisted of four basic Taekwondo techniques. Defense and counterattack with a kick comprised six defense and attack techniques. One-step sparring involved 10 movements (Supplementary Figure S1).

### 2.4. Blood Sample Analysis

After an 8 h fast, subjects were stabilized for 30 min after arrival at the laboratory, and then 10 mL of blood was drawn from the anterior vein. This was undertaken twice, before and after the 12-week Taekwondo training. The collected blood was centrifuged at 3000 rpm for 15 min to separate plasma and serum components.

ROS and antioxidant enzymes were analyzed for plasma MDA, SOD, and GPX, and inflammatory factors were evaluated for IL-6 and TNF-α. SOD activity was analyzed using a colorimetric assay using a commercial kit (706002, Cayman Chemicals, Ann Arbor, MI, USA) as previously described [19]. The serum was diluted 1:5 with a sample buffer, and 10 μL of standard and diluted samples were added to individual wells of a microtiter plate containing 200 μL of the radical detector. Thereafter, 20 μL of diluted xanthine oxidase was added into each well and mixed for a few seconds. The plate was covered with adhesive strips, and placed on a microplate shaker for 30 min at 37 °C. Finally, absorbance was measured at 450 nm using a spectrophotometer (Tecan Sunrise, TECAN GmbH, Salzburg, Austria). Plasma MDA (MBS263626, MyBioSource, San Diego, CA, USA), GPX (CSB-EL009866HU, CUSABIO, Wuhan, China), IL-6 (DY206-05, R&D Systems, Minneapolis, MN, USA), and TNF-α (DY210, R&D Systems, Minneapolis, MN, USA) levels were analyzed using a sandwich enzyme-linked immunosorbent assay as previously described [19]. Briefly, 100 µL of standards and samples were added to a microplate coated with a specific antibody and incubated for 2 h at 37 °C. Following incubation, the plate was washed three times, and 100 µL of the detection antibody was then added to each well and incubated further for 2 h at 37 °C. After washing three times, 100 µL of avidin conjugated horseradish peroxidase (HRP) was added and incubated for 2 h at 37 °C in darkness. Thereafter, washing was repeated three times, and 100 µL of substrate solution was added and incubated for 20 min at 37 °C in darkness. Finally, 50 µL stop solution was added to each well, and absorbance was read at 450 nm using a spectrophotometer.

### 2.5. Statistical Analyses

The means and standard deviations of all data were calculated using SPSS (version 22.0; IBM Corp., New York, NY, USA). Normal distribution of all data was performed using the Shapiro–Wilk test, and an independent *t*-test was used to analyze differences in the characteristics of the study subjects. A two-way repeated analysis of variance (ANOVA) was undertaken to verify the differences in times and groups for each variable. Independent and paired *t*-tests were employed to determine significant differences. All statistical significance levels were set at α = 0.05.

## 3. Results

### 3.1. Changes in Physical Fitness after 12 Weeks of Taekwondo Training Intervention

Body composition before and after the 12-week Taekwondo training intervention is summarized in Table 4. It was observed that height, weight, BMI, body fat, percent fat, and lean body mass were not significantly different between the times or the groups. Physical strength before and after the 12-week intervention is shown in Table 5. As seen from the table, physical fitness showed a significant difference across time by group interaction for the side-step (*F* = 9.333, *p* = 0.009). The post-hoc side-step test was significantly higher after the intervention than before in TSDG (*p* < 0.001). After the intervention, increases in side-step results in the TSDG were significantly larger than those in the CG (*p* < 0.001).

### 3.2. Changes in Oxidative Stress after 12 Weeks of Taekwondo Training Intervention

The levels of oxidative stress before and after the 12-week Taekwondo training intervention are shown in Table 6. Oxidative stress showed a significant difference across time by group interaction for MDA (*F* = 18.222, *p* = 0.001) and SOD (*F* = 48.297, *p* = 0.000). Following the post-hoc test, MDA was significantly lower after the intervention than before in the TSDG (*p* < 0.001). In addition, SOD was significantly higher after the intervention than before in the TSDG (*p <* 0.001). After the intervention, SOD levels in the TSDG were also significantly higher than those in the CG (*p* < 0.01). However, GPx did not significantly differ across time by group interactions.

### 3.3. Changes in Chronic Inflammatory Factors after 12 Weeks of Taekwondo Training Intervention

The levels of chronic inflammatory markers before and after the 12-week Taekwondo training intervention are depicted in Table 7. Inflammatory markers showed a significant difference across time by group interaction for TNF-α (*F* = 15.473, *p* = 0.001). Based on the post-hoc test, TNF-α was observed to be significantly lower after the intervention than before in the TSDG (*p* < 0.05). After the intervention, TNF-α levels in the TSDG were significantly lower than those in the CG (*p* < 0.05). However, IL-6 levels did not significantly differ across time by group interactions.

## 4. Discussion

The results of this study showed that the 12-week Taekwondo self-defense training intervention positively improved physical fitness and was effective in modifying oxidative stress and levels of inflammation in postmenopausal women. The findings of this study support the results of a systematic review that concluded that Olympic combat sports, including Taekwondo, improved the physical-functional, physiological, and psycho-emotional health of the elderly population [20].

Fat accumulation increases with age, owing to a rapid decline in estrogen levels in middle-aged women [21]. Accelerated obesity in women generally leads to a decrease in physical activity as well as fitness levels, resulting in metabolic syndrome, the prevalence of which is more than twice that in men [22]. Regular aerobic exercise has been shown to reduce the risk of metabolic syndrome by positively altering body composition and blood lipid profiles in middle-aged women. A previous study reported that 12 weeks of regular exercise decrease body weight, percentage of body fat, and TG levels and increase high-density lipoprotein cholesterol in middle-aged women [23]. Another report documented that a 12-week Taekwondo training program conducted three times per week significantly decreased body weight, BMI, and resting heart rate in post-menopausal women with stage 2 hypertension [15], indicating that regular Taekwondo training also has a positive effect on the body composition of middle-aged and elderly women with age-related diseases. Participants in this study were healthy postmenopausal women who were relatively younger than those in other studies and whose body composition was also within the normal range. These participant characteristics partially explain why there was no change in body composition in both the control and exercise groups. Body composition also may have not been significantly altered after the Taekwondo intervention owing to other variables such as physical activity, dietary intake, and supplement intake, which were not accounted for in the study. Therefore, analyses of various subjects of different ages, body compositions, and medical history will be needed to clearly investigate the effect of Taekwondo training on the body composition of postmenopausal women.

The American College of Sports Medicine defines physical fitness as “a set of attributes or characteristics individuals have or achieve that relates to their ability to perform physical activity” [24]. In particular, physical fitness is necessary for physical activity and is also an essential element in other health aspects, such as prevention of falls, osteoporosis, and fractures [25,26]. In this study, agility statistically improved after 12 weeks of Taekwondo self-defense training. Muscle strength, quickness, and flexibility tended to improve but the difference was not significant. When comparing available studies related to Taekwondo interventions, participation in Taekwondo training for 16 weeks was reported to significantly improve muscle strength, flexibility, and aerobic endurance levels in elderly women [14]. Another systematic review concluded that Taekwondo training can lead to an improvement in body composition (fat loss) and physical fitness (flexibility) [27]. Though these results contradict the results of this study, it may be worthwhile to note that the difference may have been due to different training methods employed (Taekwondo self-defense training vs. traditional Taekwondo training). Unfortunately, although there was no significant difference in physical fitness at baseline, it tended to be higher in the TSDG group than in the CG group. This is speculated to be due to exclusion of results of four participants who did not complete the 12-week taekwondo training and had to be excluded. Nevertheless, it is significant that the side-step of the TSDG group increased after the training compared to that before it. The Taekwondo self-defense training program used in this study was composed of movements designed to cope with emergencies, and repetitive performance induced improvement in agility. Therefore, the training program apparently helped perform side-step, which was a method of measuring fitness in this study.

MDA is a representative ROS and is produced in response to damage to polyunsaturated fatty acids in the muscle membrane during the process of transporting oxygen to active muscles and excess use of oxygen by the muscles while exercising [28]. Meanwhile, to prevent oxidative damage from reactive oxygen compounds produced during metabolic or other processes, the antioxidant defense system operates as a defense mechanism for protecting the body [29]. SOD, an antioxidative compound in the human body, is a very important enzyme that copes with homeostatic disruptions and oxidative stress caused by exercise and acts as the first defense against tissue damage by reducing ROS [30]. Regular exercise has positive effects on reducing ROS levels by enhancing the activation of antioxidant systems [31]. In this study, MDA significantly decreased and SOD increased in the TSDG after 12 weeks of Taekwondo self-defense training intervention. These results are consistent with those of a previous study reporting that 12 weeks of aerobic exercise for men and women, aged 65–80 years, had a positive effect on the activation of antioxidant enzymes [32]. In another study, folk dancing with elastic band exercise that was conducted in 12 elderly women aged 65–75 years for 12 weeks had a positive effect on blood MDA concentration as well as activity of antioxidant enzymes such as SOD and GPx [33]. In addition, a 8-week Tai Chi training (a traditional Chinese martial art) increased erythrocyte GPX activity and total plasma antioxidant status in pre- and post-menopausal sedentary women [34]. Additionally, in a study involving 16 weeks of Taekwondo exercise in 20 overweight and obese adolescents, the SOD level was higher and the MDA level significantly lower in the Taekwondo exercise group than in the CG [35]. This reduction in oxidative stress induced as a result of physical activity and exercise as reported in previous studies is similar to our observations, since the exercise intensity of Taekwondo self-defense training in this study was at HRR 40–70%, which is within the range of effective moderate-intensity exercise. Therefore, the intensity of Taekwondo self-defense training can be considered to be similar to aerobic training, as results are reproducible without a large load (resistance) [36].

As mentioned earlier, after menopause, there is a rapid decrease in estrogen secretion, and an increase in fat cells and in body inflammatory reactions, which in turn results in an increased risk of developing metabolic syndrome. Popko et al. reported that obesity is closely related to the level of pro-inflammatory cytokines, and increased inflammatory cytokines such as IL-6 and TNF-αn lead to the persistence of inflammation in obese individuals [37]. IL-6 and TNF-α are representative inflammatory cytokines that are mainly produced by activated macrophages and secreted by activated lymphocytes, endothelial cells, and fibroblasts [38]. TNF-α is secreted from fat and muscle tissues and induces inflammation in blood vessels, and also produces and secretes molecules related to fat storage [39]. Therefore, in postmenopausal women, though estrogen levels decrease, there is an increase in mast cells and pro-inflammatory factors that cause inflammatory reactions in the body, resulting in obesity and aging. As such, postmenopausal women are always at risk of inflammatory reactions.

Representative effects of regular exercise include reduction of inflammatory responses and enhancement of antioxidant function. Inflammatory factors such as IL-6 and C-reactive protein levels have been shown to decrease after long-term exercise training in chronic cardiovascular disease patients, the elderly, the sedentary, and healthy individuals [40,41]. In addition, in a previous study that compared the effects of aerobic exercise and resistance exercise, TNF-α, IL-6, and BMI decreased after both exercise interventions. However, the authors concluded that aerobic exercise is more appropriate in modulating pro-inflammatory cytokines and improving quality of life in postmenopausal obese women [42]. In the present study, TNF-α in TSDG was reduced after 12 weeks of Taekwondo self-defense training intervention, and these results are consistent with the results of previous studies, confirming that regular exercise alleviates inflammatory responses in the blood. Although IL-6 showed a decreasing trend following the training, the difference was not significant. A possible reason for this may be the fact that IL-6 was analyzed in middle-aged women who did not have an underlying medical condition. Further studies are warranted to determine the effect of Taekwondo self-defense training on the improvement of inflammatory factors in different groups of individuals.

## 5. Conclusions

The 12-week Taekwondo self-defense training intervention improved agility and decreased oxidative stress and inflammatory responses in postmenopausal women. The results of this study suggest that Taekwondo self-defense training is an effective exercise for improving agility while providing physiological benefits for postmenopausal women.

## Figures and Tables

**Table 1:** Characteristics of subjects.

Variable	CG (*n* = 8)	TSDG (*n* = 8)	*t*	*p*
Age (year)	50.25 ± 2.12	49.62 ± 4.03	0.388	0.706
Height (cm)	160.67 ± 6.12	158.98 ± 2.67	0.715	0.487
Weight (kg)	60.10 ± 6.70	58.92 ± 9.05	0.295	0.772
BMI (kg/m^2^)	23.05 ± 1.32	23.36 ± 3.89	−0.215	0.835
Fat mass (kg)	19.46 ± 3.39	17.93 ± 5.90	0.633	0.537
Percent fat (%)	32.31 ± 4.02	29.76 ± 5.32	1.146	0.271
LBM (kg)	40.53 ± 4.67	40.98 ± 3.27	−0.223	0.827

Data are presented as the mean ± standard deviation. CG, control group; TSDG, Taekwondo self-defense training group; BMI, body mass index; LBM, lean body mass.

**Table 2:** Measuring equipment for each physical fitness component.

Component	Manufacturer	Equipment
Body composition	InBody (Seoul, Korea)	InBody J05
Lower back strength	Takei Scientific Instruments (Niigata, Japan)	T.K.K.5402
Side-step	CASIO (Tokyo, Japan)	DPS2750
Trunk flexion	Takei Scientific Instruments (Niigata, Japan)	T.K.K.5103
Sargent jump	Takei Scientific Instruments (Niigata, Japan)	T.K.K.5406

**Table 3:** Twelve-week Taekwondo self-defense training program.

	Taekwondo Self-Defense Training	
Warm-up (10 min)	Static stretching	
Main exercise (40 min)	Taekwondo self-defense <Basic movement> five times each side, 1–2 min rest	(1) Chigi (Strike) (2) Jireugi (Punch) (3) Chagi (Kick) (4) Makgi (Block)
	Taekwondo self-defense <Defense and counterattack with a kick> five times, 1–2 min rest	(1) Olgul Oreunbal Apdollyeo-Chagi (Right foot front spin kick to the face) (2) Olgul Oenbal Yeop-Chagi (Left foot side kick to the face) (3) Olgul Oreunbal Yeop-Chagi (Right foot side kick to the face) (4) Dwidola Yeop-Chagi (Turning side kick to the flank) (5) Apdollyeo-Chagi (Front foot spin kick to the face) (6) Ttwimyeo Yeop-Chagi(Jump and side kick to the flank)
	Taekwondo self-defense <one-step sparring> five times	(1~10) No.1~No.10 Gyeorugi (10 indicated movements)
Cool-down (10 min)	Static stretching	

Intensity: 50–70% of the heart rate reserve; frequency: four times/week (Monday, Tuesday, Thursday, Friday).

**Table 4:** Changes in body composition after the 12-week Taekwondo training intervention.

Variable		Baseline	12 Weeks	Group × Times *F p*	
Height (cm)	CG	160.67 ± 6.12	160.95 ± 6.27	0.575	0.461
	TSDG	158.98 ± 2.67	159.41 ± 2.74		
Weight (kg)	CG	60.10 ± 6.70	60.26 ± 6.65	0.070	0.796
	TSDG	58.92 ± 9.05	59.18 ± 9.19		
BMI (kg/m^2^)	CG	23.05 ± 1.32	23.20 ± 1.34	0.068	0.798
	TSDG	23.36 ± 3.89	23.34 ± 3.99		
Body fat (kg)	CG	19.46 ± 3.39	19.55 ± 3.56	0.242	0.630
	TSDG	17.93 ± 5.90	18.15 ± 6.03		
Percent fat (%)	CG	32.31 ± 4.02	32.51 ± 4.36	0.230	0.639
	TSDG	29.76 ± 5.32	28.38 ± 5.24		
LBM (kg)	CG	40.53 ± 4.67	40.67 ± 4.81	0.083	0.778
	TSDG	40.98 ± 3.27	41.03 ± 3.30		

Data are presented as mean ± standard deviation. BMI: body mass index, LBM: Lean body mass. CG: Control group, TSDG: Taekwondo self-defense training group.

**Table 5:** Changes in physical fitness after the 12-week Taekwondo training intervention.

Variable		Baseline	12 Weeks	Group × Times *F p*	
Lower back strength (kg)	CG	44.35 ± 6.25	43.83 ± 5.94	0.864	0.368
	TSDG	59.18 ± 22.09	62.06 ± 16.69		
Trunk flexion (cm)	CG	19.70 ± 2.85	19.32 ± 2.83	0.020	0.890
	TSDG	21.90 ± 5.30	21.47 ± 5.33		
Side-step (*n*)	CG	5.37 ± 0.51	5.37 ± 0.51	9.333	0.009 *
	TSDG	7.37 ± 1.06 ^#^	8.37 ± 1.06 ^#,$^		
Sargent jump (cm)	CG	25.87 ± 6.19	24.62 ± 6.18	2.132	0.166
	TSDG	26.75 ± 4.23	27.12 ± 4.32		

Data are presented as mean ± standard deviation. CG, control group; TSDG, Taekwondo self-defense training group * *p* < 0.05; significant main effect and/or interaction. ^#^ *p* < 0.05; significant difference between CG and TSDG. ^$^ *p* < *0*.05; significant difference between before and after the 12-week Taekwondo training intervention.

**Table 6:** Changes in oxidative stress after the 12-week Taekwondo training intervention.

Variable		Baseline	12 Weeks	Group × Times *F p*	
MDA (nmol/mL)	CG	2.21 ± 0.49	2.51 ± 0.87	18.222	0.001 *
	TSDG	2.60 ± 0.67	1.91 ± 0.34 ^$^		
SOD (U/mL)	CG	2.27 ± 0.81	2.04 ± 0.47	48.297	0.000 *
	TSDG	1.49 ± 0.34	3.42 ± 0.92 ^#,$^		
GPx (μU/mL)	CG	34.38 ± 11.70	33.86 ± 10.48	0.491	0.495
	TSDG	36.35 ± 11.23	37.66 ± 10.86		

Data are presented as mean ± standard deviation. MDA, malondialdehyde; SOD, superoxide dismutase; GPx, glutathione peroxidase. CG, control Group; TSDG, Taekwondo self-defense training group * *p* < 0.05; significant main effect and/or interaction. ^#^ *p* < 0.01; significant difference between CG and TSDG. ^$^ *p* < 0.001; significant difference between before and after the 12-week Taekwondo training intervention.

**Table 7:** Changes in inflammatory factors after the 12-week Taekwondo training intervention.

Variable		Baseline	12 Weeks	Group × Times *F p*	
TNF-α (pg/mL)	CG	4.69 ± 0.58	4.79 ± 0.50	15.473	0.001 *
	TSDG	4.55 ± 0.65	4.09 ± 0.60 ^#,$^		
IL-6 (pg/mL)	CG	3.95 ± 0.76	4.10 ± 0.65	4.310	0.057
	TSDG	4.02 ± 0.78	3.66 ± 0.42		

Data are presented as mean ± standard deviation. TNF-α: Tumor necrosis factor-alpha; IL-6: Interleukin-6. CG, control group, TSTG, Taekwondo self-defense training group * *p* < 0.05; significant main effect and/or interaction. ^#^ *p* < 0.05; significant difference between CG and TSDG. ^$^ *p* < 0.05; significant difference between before and after the 12-week Taekwondo training intervention.

## Data Availability

Data generated and analyzed during this study are included in this article. Additional data are available from the corresponding author on request.

## Supplementary Materials

The following are available online at https://www.mdpi.com/article/10.3390/healthcare9080985/s1: Figure S1: Taekwondo self-defense training program (one-step sparring).
