# Supplementary material for: Effect of Regular Taekwondo Self-Defense Training on Oxidative Stress and Inflammation Markers in Postmenopausal Women
Source: Healthcare (Basel). 2021 Aug 3;9(8):985. doi: 10.3390/healthcare9080985 (PMC8394346; doi:10.3390/healthcare9080985)
Supplement: Supplementary file 1 [file healthcare-09-00985-s001.zip › healthcare-1254927-supplementary.pdf]

Taekwondo self-defense training program consists of 10 promised movements (No.1–10). The specific movement and description are as follows.

No.1		No.2	
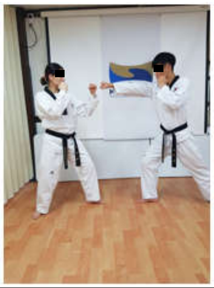	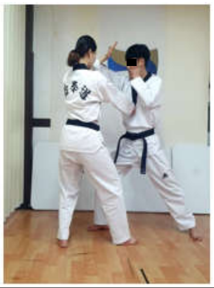	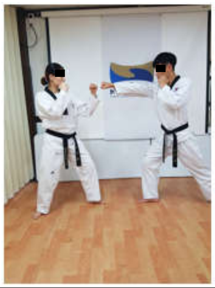	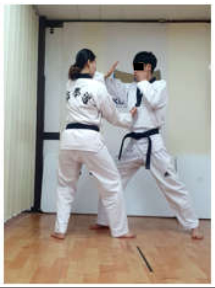
Ready	Right sidestep<br>Left hand knife outside block<br>Middle punch	Ready	Right sidestep<br>Left hand knife outside block<br>Middle turn over punch

No.3		No.4	
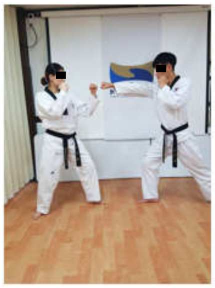	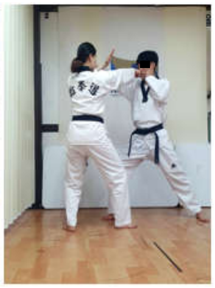	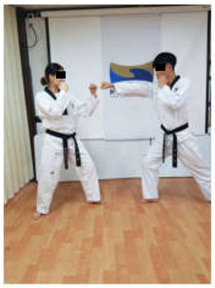	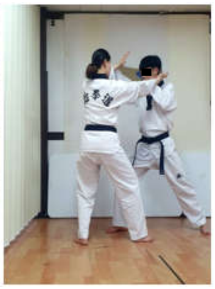
Ready	Right sidestep<br>Left hand knife outside block<br>Upper punch	Ready	Right sidestep<br>Left hand knife outside block<br>Neck hand knife inside strike

No.5		No.6	
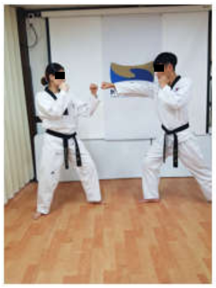	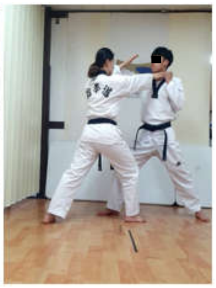	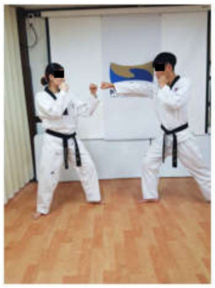	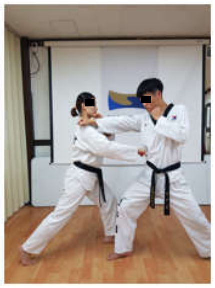

Ready	Right sidestep<br>Left hand knife outside block Forward strike to the neck		Right sidestep<br>Left hand palm heel block<br>Middle punch

No.7		No.8	
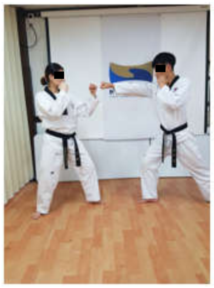	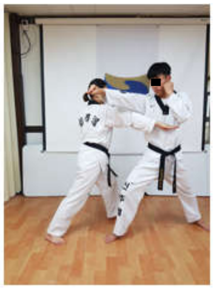	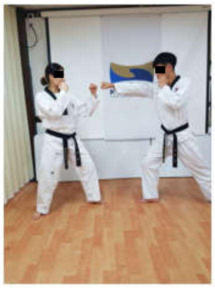	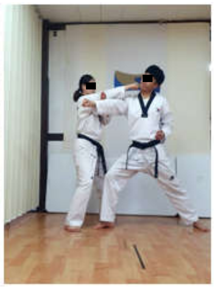
Ready	Right sidestep<br>Left hand palm heel block<br>Right ridge hand strike	Ready	Right sidestep<br>Left hand palm heel block<br>Upper punch

No.9		No.10	
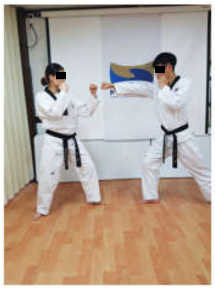	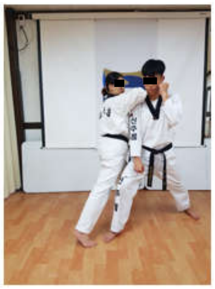	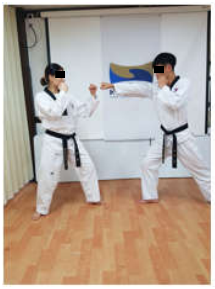	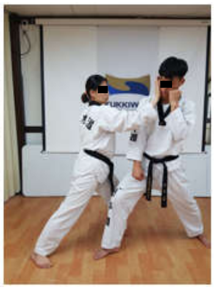
Ready	Right sidestep<br>Left hand palm heel pressing block<br>Elbow turning strike to the face	Ready	Right sidestep<br>Left hand palm heel pressing block<br>Palm heel forward strike to the chin

Figure S1. Taekwondo self-defense training program (one-step sparring).
